# Effectiveness Of A Staff Training Program To Stimulate Physical Activity In Homecare: A Cluster RCT

**DOI:** 10.1093/geroni/igab046.3479

**Published:** 2021-12-17

**Authors:** Silke Metzelthin, Teuni H Rooijackers, G A Rixt Zijlstra, Erik van Rossum, Annemarie Koster, Silvia Evers, Valeria Lima Passos, Gertrudis I J M Kempen

**Affiliations:** 1 Maastricht University, Maastricht, Limburg, Netherlands; 2 Zuyd University of Applied Sciences, Maastricht, Limburg, Netherlands; 3 m, Maastricht, Limburg, Netherlands

## Abstract

Reablement encourages older adults to do things themselves rather than having things done for them. To implement reablement in practice homecare staff needs the right knowledge, attitude, skills and support. This study evaluated the effectiveness of the “Stay Active at Home” reablement training program. A 12-month cluster-RCT was conducted, involving staff (n=313) and clients (n=264) from 10 homecare teams, five of which were trained. Effects were evaluated using data from accelerometers, physical performance tests, questionnaires and electronic patient records. No beneficial effects were observed in older adults for sedentary behavior; daily, physical, and psychological functioning; and falls. In homecare staff there were no statistically significant differences between study groups for self-efficacy and outcome expectations scores except for higher self-efficacy scores in more compliant staff (adjusted mean difference: 1.9 [95% CI 0.1, 3.7]). No differences were observed for any cost category except for domestic help costs in the intervention group (adjusted mean difference: €-173 [95% CI -299, -50]). The probability that “Stay Active at Home” is cost-effective compared to usual care at a willingness-to-pay of €20,000 was 19.7%/ daily minute of sedentary time averted, 19.2%/ percent of sedentary time averted as proportion of wake/wear time, and 5.9%/QALY gained, respectively. The reablement training program needs further development based on the lessons learned before wider implementation.

